# Efficacy and safety of low-molecular-weight heparin in patients with sepsis: a meta-analysis of randomized controlled trials

**DOI:** 10.1038/srep25984

**Published:** 2016-05-16

**Authors:** Yu Fan, Menglin Jiang, Dandan Gong, Chen Zou

**Affiliations:** 1 Institute of Molecular Biology & Translational Medicine, the Affiliated People’s Hospital, Jiangsu University, Zhenjiang, Jiangsu, PR China (212002); 2 Department of general surgery, the Affiliated People’s Hospital, Jiangsu University, Zhenjiang, Jiangsu, PR China (212002)

## Abstract

Low-molecular-weight heparin (LMWH) is part of standard supportive care. We conducted a meta-analysis to investigate the efficacy and safety of LMWH in septic patients. We searched Pubmed, Embase, CKNI and Wanfang database prior to July 2015 for randomized controlled trials investigating treatment with LMWH in septic patients. We identified 11 trials involving 594 septic patients. Meta-analysis showed that LMWH significantly reduced prothrombin time (mean differences [MD] −0.88; 95% CI −1.47 to −0.29), APACHE II score (MD −2.50; 95% CI −3.55 to −1.46), and 28-day mortality (risk ratio [RR] 0.72; 95% CI 0.57–0.91) as well as increased the platelet counts (MD 18.33; 95% CI 0.73–35.93) than the usual treatment. However, LMWH did not reduce D-dimer (MD −0.34; 95% CI −0.85 to 0.18). LMWH also significantly increased the bleeding events (RR 3.82; 95% CI 1.81–8.08). LMWH appears to reduce 28-day mortality and APACHE II score among septic patients. Bleeding complications should be monitored during the LMWH treatment. As for limited data about LMWH and sepsis in the English literature, only trials published in the Chinese were included in the meta-analysis.

Sepsis is a clinical syndrome of microbial infection complicated by systemic inflammation. Sepsis remains a major public health problem. The incidence of severe sepsis is estimated up to 100 cases per 100 000 population^1^. Despite advances in intensive care technologies, the hospital mortality rate of severe sepsis was 48.7% in China^2^. Sepsis can induce coagulatory activation, which may contribute to deteriorate organ function^3^,^4^. Cross talk exists between inflammatory and coagulation system^5^. Therefore, targeting the hypercoagulable state should be a promising approach in the treatment of sepsis.

The survival benefits of coagulation inhibitors, recombinant human activated protein C as an adjunctive therapy for sepsis have been demonstrated^6^,^7^. However, the high cost and bleeding risk limit its application for the majority of patients. Heparin has the potential role in the treatment of sepsis because of similar anticoagulant actions^8^. Heparin can be divided into unfractionated and low-molecular-weight heparin(LMWH)^9^. Unfractionated heparin is the most commonly used anticoagulant worldwide. LMWH is an attractive alternative anticoagulant due to its superior antithrombotic efficacy and fewer reported bleeding risk^10^. Several studies^11^,^12^,^13^,^14^,^15^,^16^,^17^,^18^,^19^,^20^,^21^ have been published in the literature evaluating the effects of LMWH in sepsis. In general, findings from these studies suggested that LMWH might be useful in these septic patients. However, small sample size of each study might lack statistical power to draw definitive conclusions.

Here, we conducted a meta-analysis using the available randomized control trials (RCTs) to evaluate the efficacy and safety of LMWH treatment in septic patients.

## Results

### Search results and baseline characteristics

**Figure 1:**
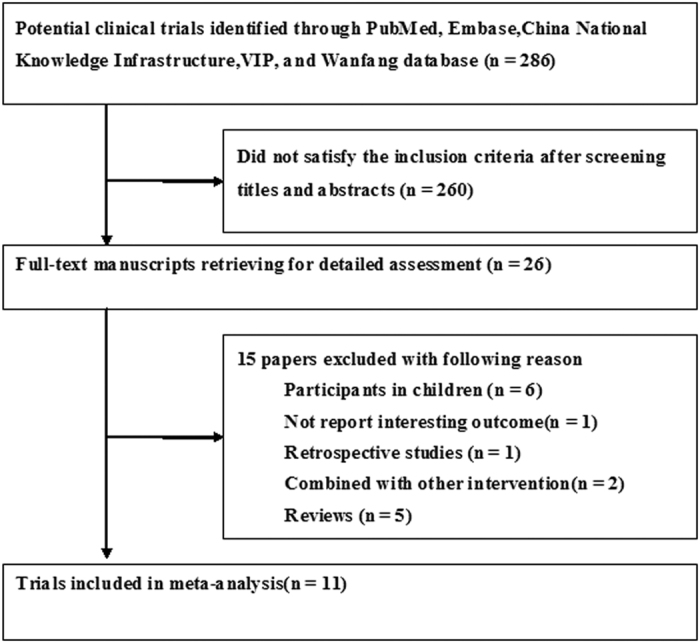
Diagram of trials’ selection process.

**Figure 2:**
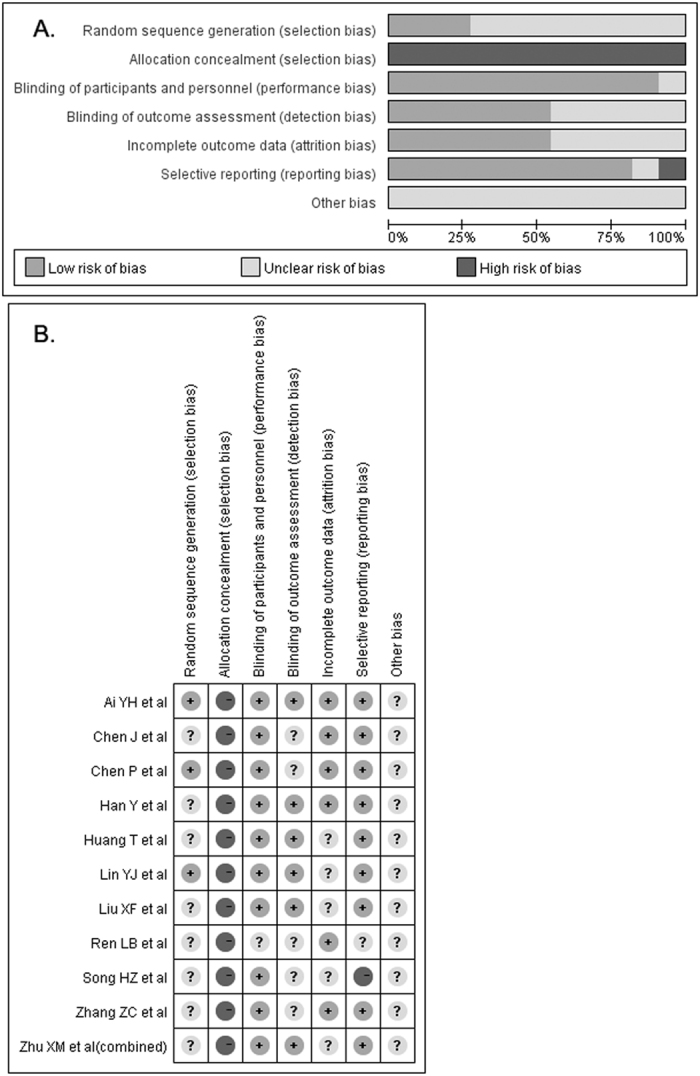
Risk of bias graph (**A**) and risk of bias summary (**B**).

Our preliminary literature search yielded 286 potential articles, of which 260 were excluded after screening titles and abstracts. After retrieving for full-text manuscript, 15 citations were excluded for various reasons. Finally, 11 trials^11^,^12^,^13^,^14^,^15^,^16^,^17^,^18^,^19^,^20^,^21^ were eligible for the meta-analysis (Fig. 1). Baseline characteristics of the trials are listed in Table 1. All the trials were performed in China and published in Chinese. Eleven trials involved 594 patients with 329 in LMWH groups and 265 in the usual treatment groups. Patients in LMWH groups received two types of heparin: low-molecular weight heparin calcium and low-molecular weight heparin sodium. LMWH treatment durations were 7 and 14 days. Ten trials^11^,^12^,^13^,^15^,^16^,^17^,^18^,^19^,^20^,^21^ determined the circulating levels of prothrombin time, platelet, d-dimer, pro-inflammatory cytokines interleukin-6 (IL-6), tumor necrosis factor-α (TNF-α), and clinical acute physiology and chronic health evaluationII (APACHE II) score after 7 days’ LMWH treatment, one trial^14^ measured above outcomes after 14 days’ treatment. The sample size ranged from 18 to 105. In general, the included trials were usually classified as low to moderate quality. The risk of bias of each trial is summarized in Fig. 2.

### Clinical outcomes

**Figure 3:**
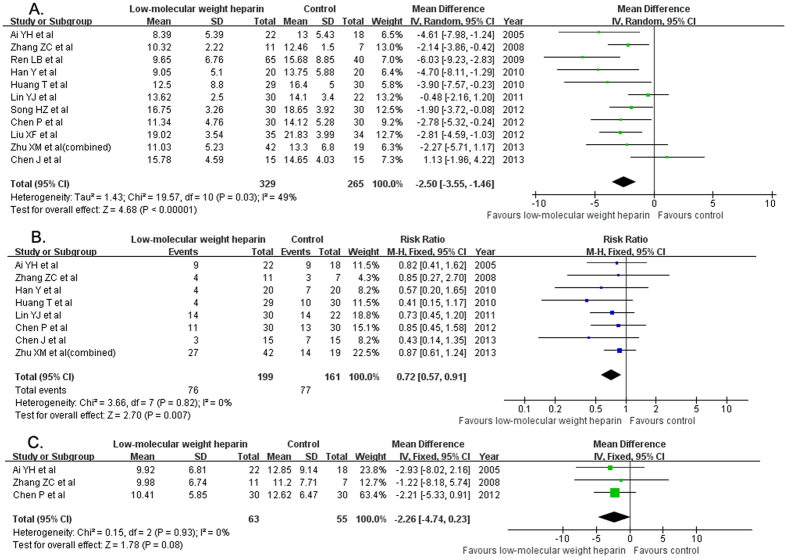
Forest plots showing the clinical outcome of the eligible trials comparing low-molecular weight heparin to the usual treatment. APACHE II score (**A**); 28-day mortality (**B**); length of intensive care unit stay (**C**).

All the included trials provided the changes of APACHE II score. Treatment with LMWH significantly reduced APACHE II score (MD −2.50; 95% CI −3.55 to −1.46; I^2^ = 49%, P < 0.03) compared with the usual treatment group in a random effect model (Fig. 3A). Eight trials^11^,^12^,^14^,^15^,^16^,^18^,^20^,^21^ reported the 28-day mortality outcomes. Treatment with LMWH significantly reduced 28-day mortality (RR 0.72; 95% CI 0.57–0.91; I^2^ = 0%, P = 0.82) than the usual treatment group in a random effect model (Fig. 3B). There was no significant difference in the length of intensive care unit (ICU) stay (MD −2.26 days; 95% CI −4.74–0.23; I^2^ = 0%, P = 0.93) between the two groups (Fig. 2C) in three trials^11^,^12^,^18^.

### Changes of serum pro-inflammatory cytokines

**Figure 4:**
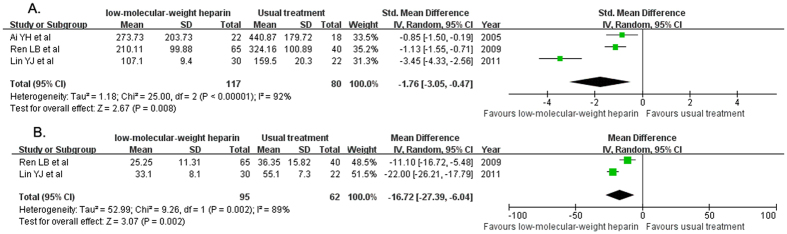
Forest plots showing changes of serum pro-inflammatory cytokines of the eligible trials comparing low-molecular weight heparin to the usual treatment. Interleukin-6 (**A**); tumor necrosis factor-α (**B**).

Serum IL-6 level changes were available in three trials^11^,^13^,^16^. Treatment with LMWH significantly reduced serum IL-6 level (SMD −1.76; 95% CI −3.05 to −0.47; I^2^ = 92%, P < 0.00001) compared with the usual treatment group in a random effect model (Fig. 4A). Serum TNF-α level was reported in two trials^13^,^16^. Treatment with LMWH significantly reduced serum TNF-α level (MD −16.72; 95% CI −27.39 to −6.04; I^2^ = 89%, P = 0.002) compared with the usual treatment group in a random effect model (Fig. 4B).

### Changes of anticoagulant activity

**Figure 5:**
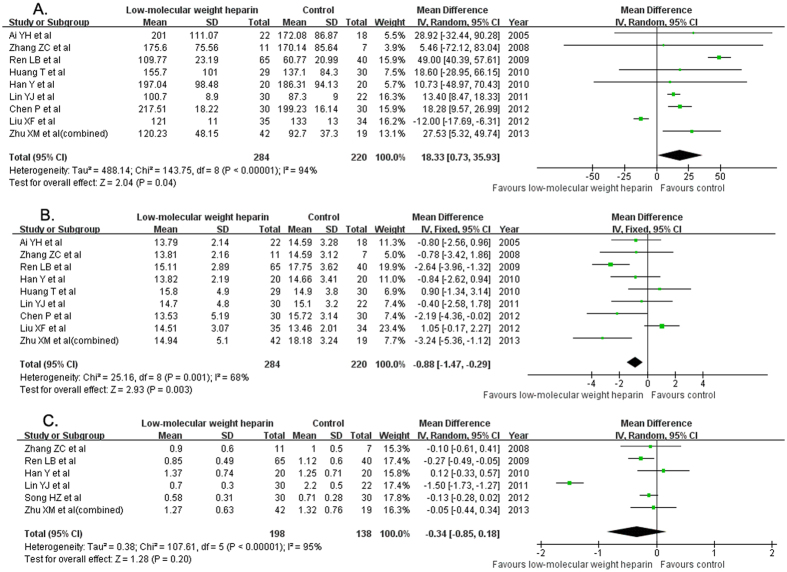
Forest plots showing changes of anticoagulant activity of the eligible trials comparing low-molecular weight heparin to the usual treatment. Platelet counts (**A**); prothrombin time (**B**); D-dimer (**C**).

Nine trials^11^,^12^,^13^,^14^,^15^,^16^,^18^,^19^,^20^ provided the changes of platelet counts. Treatment with LMWH significantly increased platelet counts (MD 18.33; 95% CI 0.73–35.93; I^2^  = 94%, P < 0.001) than the usual treatment group in a random model (Fig. 5A). Nine trials^11^,^12^,^13^,^14^,^15^,^16^,^18^,^19^,^20^ provided the changes of prothrombin time. LMWH treatment significantly reduced PT (MD −0.88; 95% CI −1.47 to −0.29; I^2^ = 68%, P = 0.003) than the usual treatment group in a random model (Fig. 5B). Six trials^12^,^13^,^15^,^16^,^17^,^20^ reported the changes of serum D-dimer. There was no significant difference in the changes of D-dimer (MD −0.34; 95% CI −0.85 −0.18; I^2^ = 95%, P < 0.001) than the usual treatment group in a random model (Fig. 5C).

### Bleeding complications

**Figure 6:**
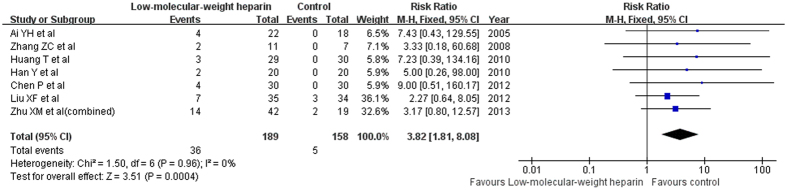
Forest plots showing risk of total bleeding complications comparing low-molecular weight heparin to the usual treatment.

Seven trials^11^,^12^,^14^,^15^,^18^,^19^,^20^ reported bleeding events. Of the total bleeding events, the frequency of tracheal intubation and deep vein puncture or injection site bleeding occurred 20 (6.07%) of 329 patient receiving LMWH, while frequency in the control group was 3 (1.13%) of 265 patients. Gastrointestinal hemorrhage occurred 14 (4.26%) of 329 patient receiving LMWH, while frequency in the control group was 2 (0.75%) of 265 patients. In addition, one case of small ecchymoses^19^ and gum bleeding^20^ was also noted. When we pooled total bleeding events from seven studies, the risk of any bleeding events was significantly increased to 3.82 (95% CI 1.81–8.08; I^2^ = 0%, P = 0.96) in a fixed effect model (Fig. 6).

### Subgroup analyses, sensitivity analyses, and publication bias

Subgroup analyses and publication bias were conducted based on changes in APACHE II score and 28-day mortality. The results of subgroup analyses were presented in Table 2.

Sensitivity analyses were performed by sequentially omitting one study at each turn. The results showed there was no change in the direction of effect sizes in APACHE II score and 28-day mortality outcomes. Evidence of publication bias for trial reporting the changes of APACHE II score was not observed by the Begg’s test (P = 0.119) and Egger’s test (P = 0.141). Potential publication bias for trial reporting 28-day mortality was observed in the Egger’s test (P = 0.041) but not in the Begg’s test (P = 0.266).

## Discussion

Our meta-analysis suggests that treatment with LMWH appears to reduce 28-day mortality and APACHE II score, as well as improvement in pro-inflammatory cytokines and anticoagulant activity among septic patients. Increased any bleeding complications with LMWH administration are identified. However, these findings were concluded by the trials with methodological flaws, and recommendation of these findings should be cautioned.

Sepsis has been grouped into sepsis, severe sepsis, followed by the presence of multiorgan dysfunction, and septic shock^22^. Sepsis-related inflammation may activate the coagulation system, consume multiple clotting factors and resulting in disseminated intravascular coagulation (DIC)^23^,^24^. The Japanese guidelines supported the aggressive treatment of septic DIC^25^,^26^. In contrast, the guidelines of the Surviving Sepsis Campaign of the European Union and the USA did not recommend treatment for septic DIC^27^,^28^. Two previous systematic reviews and meta-analyses^29^,^30^ showed that heparin therapy may be associated with decreased mortality in patients with sepsis. However, these two studies mainly focused on the unfractioned heparin but not LMWH. Therefore, the efficacy and safety of LMWH for sepsis is not well established.

Severe sepsis is associated with higher levels of pro-inflammatory markers. The massive inflammatory activation played a key role in the development of sepsis to septic shock^31^. Of these proinflammatory cytokines, IL-6 and TNF-α, have got the most attention. Higher serum IL-6 or TNF-α levels indicate an increased risk of mortality^32^. In the current study, adjuvant treatment with LMWH reduced serum IL-6, TNF-α level to some extent which indicated LMWH had the potential to inhibit the inflammatory process. The potential anti-inflammatory property of LMWH might partly explain its beneficial mechanism.

Cytokine production contributes to the activation of platelets. Coagulation abnormalities are frequent complications of sepsis^33^. Manifestation coagulation abnormalities in sepsis range from a decrease in platelet counts and subclinical prolongation of prothrombin time to DIC^34^. The additional benefits of adjunctive use of recombinant human activated protein C in septic patients supported anticoagulation strategy in the treatment of sepsis. LMWH may exert similar beneficial effects as recombinant human activated protein C. Our study found that LMWH could increase platelet counts and reduce prothrombin time. D-dimer is a significant prognostic factor in patients with suspected infection and sepsis^35^. However, LMWH did not significantly change d-dimer. Therefore, anti**-**platelet aggregation and anticoagulant properties are another potential pharmacological mechanism.

Benefits of adjunctive use of LMWH have been demonstrated by improvement of APACHE II score. APACHE II score is a widely accepted indicator of disease severity. There was no significant difference in baseline APACHE II score. Adjunctive treatment with LMWH significantly reduced 2.5 points of APACHE II score compared with control, which suggested application of LMWH reduced the disease severity. In addition, adjunctive use LMWH was also associated with 28% lower 28-day mortality. However, there was no significant difference in length of ICU stay. Subgroup analysis indicated high dose LMWH appeared to have more benefits in improvement in APACHE II score and 28-day mortality. Despite the beneficial effect of LMWH in septic patients, a major concern is the occurrence of bleeding complications. Our pooled results indicated that LMWH administration significantly increased bleeding events 3.82 (95% CI 1.81–8.08). LMWH treatment increased both gastrointestinal hemorrhage and invasive operation bleeding events; however, these events were mild and did not need special management. Therefore, monitoring bleeding events in the use of LMWH is recommended.

Several limitations in the current analysis should be noted. First, all the included trials conducted in China and published in Chinese; evidence of publication bias reporting 28-day mortality outcome was observed in the Egger’s test (P= 0.041). Therefore, potential publication bias cannot be ruled out. Second, the methodological quality of individual trials was mainly suboptimal, particularly the sample size of individual trials was too small. Third, significant heterogeneity was noted in pooling the anticoagulant activity and pro-inflammatory cytokines. Type of LMWH, dose or intervention duration and diverse study populations, might partly contribute to the heterogeneity. Finally, all trials included only Chinese patients, the generalization of the current findings to other ethnic population should be cautioned.

In conclusion, this meta-analysis suggests that treatment with LMWH appears to reduce 28-day mortality and APACHE II score among septic patients. The beneficial effect might be related to its anti-inflammatory and anticoagulant/antiaggregant properties. Monitoring bleeding events in the use of LMWH is recommended. More well-designed studies with a larger sample size are required before definitive recommendations can be made.

## Methods

### Literature search

This study was performed based on the Preferred Reporting Items for Systematic reviews and Meta-Analyses (PRISMA) for reporting systematic reviews and meta-analyses of RCT^36^. We searched for relevant studies using Pubmed, Embase, Cochrane Library, VIP database, China National Knowledge Infrastructure, and Wanfang database up to July 2015. A combination of the following terms was used, including heparin OR low molecular weight heparin AND sepsis OR septic shock OR septicemia OR critically ill OR systemic inflammatory response syndrome AND randomized control trials OR RCTs. The search language was limited to publications that were written in Chinese and English. References lists from eligible studies were manually searched to identify additional trials.

### Study selection

We included RCTs that used LMWH therapy in adult septic patients and reported at least prothrombin time, platelet, d-dimer, IL-6, TNF-α, APACHE II score, length of ICU stay, and 28-day mortality as outcome measures. Studies were excluded if the study designs were no RCTs or there were different regimen except for LMWH intervention between LMWH and control group. Studies were also excluded if presented a bleeding tendency because of coagulation disorders (platelet counts <30 × 10^9^), application of anticoagulant <48h prior to enrollment or serious head injury, brain arteriovenous malformation, cerebral aneurysm.

### Data extraction and methodological quality

The following items were independently extracted from each eligible studies by 2 authors (Y Fan and ML Jiang): first author name, years of publication, number and age of participant, percentage of female of participant, primary diseases distribution, and outcome measures. Any disagreements between authors were resolved through consensus. The risk of bias of each study was evaluated according to the Cochrane Handbook for Systematic Reviews of Invention^37^ on the basis of random sequence generation, allocation concealment, blinding of participants and personnel, blinding of outcome data, incomplete outcome data, and selective reporting, and others.

### Statistical analysis

All statistical analyses were conducted using the RevMan version 5.1 software. Pooled estimate was presented as mean difference (MD) or standard mean difference (SMD) with 95% confidence interval (CI) for continuous outcomes and risk ratio (RR) with 95% CI for dichotomous outcomes. Comparisons were made as LMWH plus usual treatment versus usual treatment alone. Heterogeneity of the trials was assessed using the Cochrane Q test and I^2^ statistics^38^. If I^2^ value >50% or Cochrane Q test (P < 0.10) suggested significant heterogeneity, a random effects model was selected due to considerable heterogeneity among different trials; otherwise, a fixed-effect model was used. Potential publication bias was assessed by both the Begg’s test and Egger test. Subgroup analyses were conducted according to LMWH dose (once or twice daily) and treatment duration (7 or 14 days).

## Additional Information

**How to cite this article**: Fan, Y. *et al*. Efficacy and safety of low-molecular-weight heparin in patients with sepsis: a meta-analysis of randomized controlled trials. *Sci. Rep.* **6**, 25984; doi: 10.1038/srep25984 (2016).

## Figures and Tables

**Table 1:** Characteristics of the selected trials in the meta-analysis.

Study/Year	No. patients Exp/Con	Age/Gender (% Female)	Basic APACHE II Exp/Con	Primary diseases distribution	Intervention		Outcome measures
					Experiment Group	UT (control) group	
Ai YH *et al*.^11^	22/18	42.0 ± 7.0 44.0%	14.31 ± 3.71/15.46 ± 4.61	Severe acute pancreatitis 20, suppurative cholangitis 5, multiple trauma 1, liver abscess 1, respiratory tract infection 3, and urinary tract infection 1.	Low-molecular weight heparin calcium 4100 U/12h × 7 days (SC) + UT.	Anti-infection, supplement blood capacity, stress ulcer prevention, glycemic control, nutritional support, and etiological treatment.	(1)+(2)+(3)+(6)+(7) +(8)
Zhang ZC *et al*.^12^	11/7	59.0 ± 9.0 38.9%	15.87 ± 2.21/16.41 ± 2.15	Pneumonia 9; Intestinal fistula and peritonitis 5, biliary tract infection 1, severe acute pancreatitis 1, severe trauma 1, catheter-related infection 1.	Low-molecular weight heparin calcium 6150 U/12h × 7 days (SC) + UT.	Broad spectrum antibiotics, supplement blood capacity, glycemic control, nutritional support, and etiological treatment.	(1)+(2)+(3)+(4)+(7) +(8)
Ren LB *et al*.^13^	65/40	54.26 ± 18.42 34.3%	19. 33 ± 8. 27/18. 97 ± 10. 33	NR.	Low-molecular weight heparin calcium 6150 U/12h × 7 days (SC) + UT.	Broad spectrum antibiotics, supplement blood capacity, glycemic control, nutritional support, and etiological treatment.	(1)+(4)+(5)+(6)+(7)+(8)
Huang T *et al*.^14^	29/30	61.1 ± 13.2 28.8%	24.6 ± 7.5/23.6 ± 6.1	NR.	Low-molecular weight heparin sodium 4000- 6000 U/12h × 14 days (SC) + UT.	Anti-infection, nutritional support, and etiological treatment.	(1)+(2)+(7)+(8)
Han Y *et al*.^15^	20/20	44.0 ± 8.0 45.0%	14. 80 ± 6. 08/15.15 ± 5. 33	Respiratory infection 13, suppurative cholangitis 4, multiple trauma 11, acute pancreatitis 9, skin or soft tissue infection 3.	Low-molecular weight heparin calcium 6150 U/12h × 7 days (SC) + UT.	Anti-infection and nutritional support.	(1)+(2)+(4)+(5)+(6)+(7)+(8)
Lin YJ *et al*.^16^	30/22	55.0 ± 7.0 44.2%	22.1 ± 7.0/19.3 ± 5.7	Severe acute pancreatitis 21, severe pulmonary infection 18, suppurative cholangitis 8, liver abscess 3, bacterial meningitis 1, peritonitis 1.	Low-molecular weight heparin sodium 5000 U/12h × 7 days(SC) + UT.	Broad spectrum antibiotics, fluid resuscitation, stress ulcer prevention, glycemic control, organ support, and etiological treatment.	(1)+(2)+(4)+(7)+(8)
Song HZ *et al*.^17^	30/30	73.05 ± 7.25 31.7%	21.26 ± 5.16/21.78 ± 5.38	NR.	Low-molecular weight heparin calcium 4100 U/d × 7 days (SC) + UT.	Broad spectrum antibiotics, fluid resuscitation, protecting gastric mucosa, acid suppression.	(1)+(4)
Chen P *et al*.^18^	30/30	56.5 ± 12.2 45.0%	17.52 ± 3.83/19.25 ± 4.57	Acute lung infection 15, septicemia 10, multiple trauma 8, suppurative cholangitis 7, acute pancreatitis 5, urinary tract infection 3, others 10.	Low-molecular weight heparin calcium 4100 U/d × 7 days (SC) + UT.	Broad spectrum antibiotics, fluid resuscitation, maintaining balance of water and electrolyte, and organ support.	(1)+(2)+(3)+(7)+(8)
Liu XF *et al*.^19^	35/34	56 ± 9.2 40.6%	25.12 ± 5.01/24.88 ± 4.91	Skin and soft tissue infections, severe acute pancreatitis, suppurative cholangitis, lung infection,, multiple injuries, etc.	Low-molecular weight heparin calcium 4100 U/12h × 14 days (SC)+UT.	Anti-infection, supplement blood capacity, balance of water and electrolyte., inhibition of acid secretion, nutritional support.	(1)+(7)+(8)
Zhu XM *et al*.^20^	22(A)/20(B)/19	60.5 ± 9.2 32.8%	19.8 ± 6.1/18.6 ± 4.6/17.9 ± 5.8	Lung infection 35, abdominal infection 11, biliary tract infection 8, limbs infection 4, skin and soft tissue infection 3.	A group: Low-molecular weight heparin sodium 5000 U/12h × 7 days (SC); B group 5000 U/d × 7 days (SC) + UT.	Antibiotics, supplement blood capacity, mechanical ventilation, glycemic control, nutritional support, and etiological treatment.	(1)+(2)+(4)+(7)+(8)
Chen J *et al*.^21^	15/15	64–94 43.3%	16.78 ± 5.74/15.37 ± 3.38	Chronic obstructive pulmonary disease 8, pneumonia 10, cerebral vascular disease 4, after surgery 8.	Low-molecular weight heparin calcium 6150 U/d × 7 days (SC) + UT.	Anti-infection, respiratory support, nutritional support.	(1)+(2)

Abbreviations: NR, not report; Exp, experiment; Con, control; UT, usual treatment; SC, subcutaneous injection; APACHE II, Acute physiology and chronic health evaluation II.

(1) Acute physiology and chronic health evaluation II; (2) 28-day mortality; (3) length of intensive care unit stay; (4) D-dimer; (5) tumor necrosis factor-α; (6) interleukin-6; (7) platelet; (8) prothrombin time.

**Table 2:** Subgroup analyses of APACHE II score and 28-day mortality.

Subgroup	Number of studies	Patient Number LMWH/Control	Pooled effect sizes	95% CI	Heterogeneity	
					I^2^statistic	P-value
1. APACHE II						
Heparin dose						
Once daily	4	117/94	MD-1.61	−3.12 to −0.10	57.7%	0.028
Twice daily	7	212/171	MD-3.09	−4.51 to −1.67	26.1%	0.255
Heparin type						
LMWH calcium	8	228/194	MD-2.77	−3.98 to 1.56	49.1%	0.056
LMWH sodium	3	101/71	MD-1.67	−3.68 to 0.33	36.5%	0.207
2. 28-day mortality						
Heparin dose						
Once daily	3	87/64	RR 0.78	0.57 to 1.08	0%	0.478
Twice daily	5	112/97	RR 0.67	0.47 to 0.95	0%	0.821
Heparin type						
LMWH calcium	5	98/90	RR 0.72	0.49 to 1.05	0%	0.838
LMWH sodium	3	101/71	RR 0.72	0.54 to 0.97	9.4%	0.332
Treatment duration						
7 days	7	170/131	RR 0.76	0.60 to 0.97	0%	0.917
14 days	1	29/30	RR 0.41	0.15 to 1.17	–	–

Abbreviations: LMWH, low-molecular-weight heparin; MD, mean differences; RR, risk ratio; CI, confidence interval.
